# The art of robotic colonic resection: a review of progress in the past 5 years

**DOI:** 10.1007/s13304-020-00969-2

**Published:** 2021-01-22

**Authors:** Hongyi Liu, Maolin Xu, Rong Liu, Baoqing Jia, Zhiming Zhao

**Affiliations:** 1 Department of General Surgery II, the First Medical Center of Chinese PLA General Hospital, Fuxing Road, Haidian District, Beijing, China; 2 Department of Hepatobiliary Surgery II, the First Medical Center of Chinese PLA General Hospital, Fuxing Road, Haidian District, Beijing, China

**Keywords:** Da Vinci, Robot, Colon cancer, Colectomy, Laparoscopy

## Abstract

Surgery is developing in the direction of minimal invasiveness, and robotic surgery is becoming increasingly adopted in colonic resection procedures. The ergonomic improvements of robot promote surgical performance, reduce workload for surgeons and benefit patients. Compared with laparoscopy-assisted colon surgery, the robotic approach has the advantages of shorter length of hospital stay, lower rate of conversion to open surgery, and lower rate of intraoperative complications for short-term outcomes. Synchronous robotic liver resection with colon cancer is feasible. The introduction of the da Vinci Xi System (Intuitive Surgical, Inc., Sunnyvale, CA, USA) has introduced more flexibility to colonic operations. Optimization of the suprapubic surgical approach may shorten the length of hospital stay for patients who undergo robotic colonic resection. Single-port robotic colectomy reduces the number of robotic ports for better looking and faster recovery. Intestinal anastomosis methods using totally robotic surgery result in shorter time to bowel function recovery and tolerance to a solid diet, although the operative time is longer. Indocyanine green is used as a tracer to assess blood supplementation in the anastomosis and marks lymph nodes during operation. The introduction of new surgical robots from multiple manufacturers is bound to change the landscape of robotic surgery and yield high-quality surgical outcomes. The present article reviews recent advances in robotic colonic resection over the past five years.

## Introduction

Colon cancer is one of the most common cancers worldwide and is associated with a high mortality rate [1]. The primary treatment method is colonic resection, and surgical approaches vary from open surgery to minimally invasive procedures. Laparoscopic surgery has gradually replaced laparotomy and has become an important approach in recent years. The advent of surgical robots has opened new avenues to operative techniques. The advantages of enlarged three-dimensional views, flexible wrists, and filtration of hand tremors enable surgeons to perform meticulous operations in small spaces, which has ushered in an entirely new experience to operators. The robots may afford superior ergonomic benefits and reduced workload for surgeons compared with laparoscopy [2]. Data from the National Cancer Database in the United States have demonstrated that the use of robot-assisted surgery for colon cancers is rapidly increasing, and being used more frequently in younger and healthier patients [3]. Robotic colonic resection applies not only to malignant tumors but also to some benign diseases, such as inflammatory bowel disease and colonic diverticulum. Meanwhile, innovations in surgical techniques have demonstrated clinical value, such as the suprapubic surgical approach, single-port robotic colectomy, intracorporeal anastomosis, and the use of tracers. The present article reviews recent advances in robotic colonic resection over the past 5 years.

## Materials and methods

**Fig. 1:**
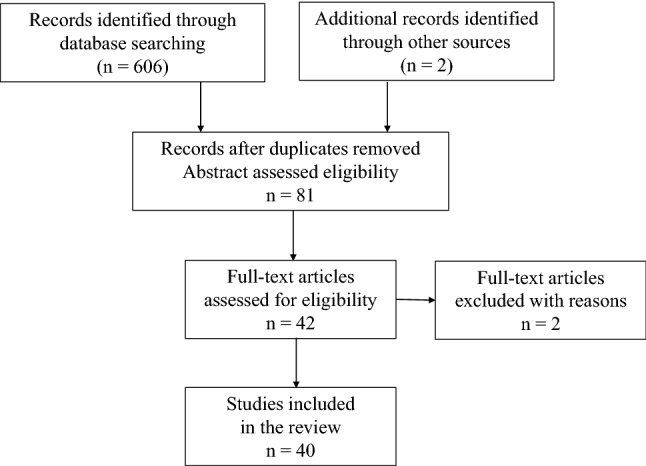
Flow-chart of papers screening

Studies were obtained from the databases Pubmed (http://www.ncbi.nlm.nih.gov/pubmed/) and Embase (http://www.embase.com) to May 15, 2020. The following text and key words were used in the search: colorectal cancer ("colorectal cancer" OR "colorectal carcinoma" OR "rectal cancer" OR "rectal carcinoma" OR "colon cancer" OR "colorectal cancer" OR "colorectal carcinoma" OR "carcinoma of colon" OR "colorectal neoplasm"), robotic surgery ("robot" OR "robotic" OR "da vinci" OR "davinci") and laparoscopic surgery ("laparoscopies" OR "laparoscopic" OR "laparoscopy"). Logical combinations of related terms were employed to maximize sensitivity. The process of papers screening was show in Fig. 1.

## Conventional controversy: robot versus laparoscopy

The efficacy of robotic colonic resection has attracted the attention of surgeons. The comparison of advantages and disadvantages of robotic versus laparoscopic surgery has always been a "hot topic". Comparative studies investigating the effects of robot-assisted (RACS) and laparoscopy-assisted colonic surgery (LACS) remain ongoing and have provided definitive data.

### Colectomy

Some studies have included patients who underwent RACS or LACS without precise distinction of tumor sites (Table 1). Estimated blood loss, rate of intraoperative blood transfusion, rate of conversion to open surgery, and rate of complications, such as ileus and anastomotic leakage, were lower using RACS than LACS in some studies, which indicated better short-term outcomes. Several studies have reported shorter time to bowel function recovery and hospital stay in the robotic group, which reflect faster post-operative recovery. In addition to these advantages, longer operative time has been described in some investigations, and higher cost of robot was reported in virtually all studies. Long-term factors, such as overall survival (OS) and disease-free survival (DFS), were comparable using both surgical approaches.

**Table 1:** Studies of clinical outcomes between robot-assisted and laparoscopy-assisted colectomy

Authors	Publication year	Study type	Period	The term	Sample size		Clinical outcomes*
					Robot	Laparoscopy	
Huerta et al. [4]	2020	Retrospective cohort	2005–2017	Short-term	105	168	Longer operative time, lower rate of ostomy creation, and similar 30-day morbidity
Chiu et al. [5]	2019	Retrospective cohort	2008–2014	Short-term	8143	174,748	Equivalent mortality rate and general medical complications
Polat et al. [6]	2019	Prospective cohort	2014.12–2017.10	Long-term	129	138	Lower conversion and intra-operative complication rate, and comparable oncological outcomes
Zhu et al. [7]	2019	Retrospective cohort	2015.7–2017.10	Short-term	104	180	Shorter postoperative recovery time of bowel function and hospital stay
Ozben et al. [8]	2019	Retrospective cohort	2010.10–2018.9	Short-term	26	56	Longer operative time and higher number of retrieved lymph nodes
Sheng et al. [9]	2018	Meta-analysis	–2017.8	Short-term	129	6749	Lower amount of blood loss, complication, mortality, bleeding, and ileus rate, shorter length of hospital stay
Pinar et al. [10]	2018	Retrospective cohort	2010.01–2015.12	Long-term	331	5647	Comparable rate of disease-free survival, all-cause mortality, and recurrence-free survival
Kulaylat et al. [11]	2018	Retrospective cohort	2013–2015	Short-term	3864	40,063	Decreased conversion rate, shorter length of hospital stay, longer operative time, and similar rate of postoperative morbidity
Nolan et al. [12]	2018	Retrospective cohort	2011–2016	Short-term	70	185	Comparable operative time and length of hospital stay
Fransgaard et al. [13]	2018	Retrospective cohort	2010.1–2015.12	Short-term	511	8104	Lower rate of conversion to laparotomy, comparable 30-day mortality and postoperative complications
Benlice et al. [14]	2017	Retrospective cohort	2013	Short-term	387	387	Longer operative time, shorter hospital stay, lower morbidity and lower rate of superficial surgical site infection, bleeding requiring transfusion, ventilator dependency, and ileus
Zhang et al. [15]	2016	Meta-analysis	2010.1–2015.10	Short-term	1466	1852	Lower estimated blood loss and length of hospital stay, similar operative time, total cost and oncological accuracy of resection

^*^Robot-assisted colectomy compared with laparoscopy-assisted colectomy

### Right hemicolectomy

Some studies have devoted close attention to patients undergoing robot-assisted (RARH) and laparoscopy-assisted right hemicolectomy (LARH) (Table 2). Similar to RACS, the advantages of RARH were primarily reflected by lower rate of conversion to open surgery and complications. Increased harvest of lymph nodes in robotic resection has been reported in several studies. Higher cost and comparable long-term outcomes of RARH versus LARH were similar to the above studies investigating colectomy.

**Table 2:** Studies of clinical outcomes between robot-assisted and laparoscopy-assisted right hemicolectomy

Authors	Publication year	Study type	Period	The term	Sample size		Clinical outcomes*
					Robot	Laparoscopy	
Waters et al. [16]	2020	Meta-analysis	1946-present	Short-term	831	3241	Reduced length of hospital stay, conversion to open surgery and incisional hernia rate, lower anastomotic complications, increased lymph node harvest, and comparable 30-day morbidity and mortality
Yozgatli et al. [17]	2019	Retrospective cohort	2015.2–2017.9	Short-term	35	64	Higher harvested lymph nodes and longer length between the vascular tie and colonic wall
Park et al. [18]	2019	Prospective randomized cohort	2009.9–2011.7	Long-term	35	36	Longer operative time and similar long-term survival
Haskins et al. [19]	2018	Retrospective cohort	2012–2014	Short-term	89	2405	Equivalent 30-day morbidity and mortality
Spinoglio et al. [20]	2018	Retrospective cohort of a prospective database	2005.10–2015.11	Long-term	101	101	Lower conversion rate and longer operative time
Kang et al. [21]	2016	Retrospective cohort	2007.6–2011	Long-term	20	43	Similar 5-year disease-free survival

^*^Robot-assisted right hemicolectomy compared with laparoscopy-assisted right hemicolectomy

### Left hemicolectomy and sigmoidectomy

Studies investigating left hemicolectomy are rare due to the low morbidity of tumors of the left semicolon. Some studies addressing benign lesions, such as diverticulum, have also reported the surgical effects (Table 3). The robot demonstrated good performance in the mobilization of colonic splenic flexure and was feasible for simple and complicated diverticular diseases of the sigmoid. Several studies reported shorter length of hospital stay, lower rate of conversion to open surgery, higher total hospital charges, and comparable postoperative complications in robotic-assisted left hemicolectomy and sigmoidectomy compared with laparoscopic surgery.

**Table 3:** Studies of clinical outcomes between robot-assisted and laparoscopy-assisted left hemicolectomy and sigmoidectomy

Authors	Publication year	Study type	Period	The term	Sample size		Clinical outcomes*
					Robot	Laparoscopy	
Alharth et al. [22]	2020	Retrospective cohort	2008–2014	Short-term	9656	187,397	Shorter length of hospital stay, higher total hospital charges and comparable postoperative complications
Bastawrous et al. [23]	2019	Retrospective cohort	2013.1–2015.9	Short-term	1301	8076	Lower rate of conversion to open surgery
Grass et al. [24]	2019	Retrospective study of a prospective database	2014–2018	Short-term	150	–	Feasible for simple and complicated diverticular diseases of sigmoid
Kim et al. [25]	2018	Retrospective cohort	2012–2017	Short-term	20	53	Dexterous dissection during the mobilization of splenic colonic flexure
Crolla et al. [26]	2018	Retrospective study of a prospective database	2012–2017	Short-term	28	–	Feasible for the resection of clinical T4 cancer of the distal sigmoid and rectum

^*^Robot-assisted left hemicolectomy and sigmoidectomy compared with laparoscopy-assisted left hemicolectomy and sigmoidectomy

### Synchronous colonic resection with liver metastases

Liver metastases are particular focus of surgeons in improving the survival of patients with colon cancer. Unfortunately, only 25% of colon cancer patients with liver metastases are suitable candidates for liver resection [27]. Dwyer et al. [28] described synchronous robotic surgery for patients with stage IV colorectal cancer with liver metastases. This retrospective review of prospectively collected data, however, included only six patients. Liver treatment was performed first in consideration of intraoperative bleeding risk. The authors reported that the robotic approach contributed to low blood loss (150–1000 mL), appropriate length of hospital stay (3–10 days), and no 30-day mortality. This study supported the potential benefits of synchronous robotic liver resection with colon cancer.

It is clear from the above studies investigating different surgical methods and tumor sites that most reported shorter length of hospital stay, lower rate of conversion to open surgery, and lower rate of intraoperative complications in robotic colonic resection, while long-term indexes in all studies, such as OS and DFS, were not significantly different from the laparoscopic approach (Table 4). The high cost of robotic surgery, mainly coming from high selling price, expensive consumables and daily maintenance expense, however, has become an important factor that restricts its application in surgery. The surgical robot, appeared as an advanced tool, do bring some benefits to patients though the long-term efficacy is comparable with laparoscopy, and studies with multicenter and long-term follow-up are needed.

**Table 4:** Comparison of robotic vs laparoscopic approach for each marker discussed regarding colonic interventions

Marker	Robotic approach	Laparoscopic approach
Rate of conversion	Lower	Higher
Operative time	Longer	Shorter
Rate of ostomy creation	Lower	Higher
Postoperative recovery time of bowel function	Shorter	Longer
Postoperative recovery time of hospital stay	Shorter	Longer
30-day morbidity	Lower	Higher
Cost	Higher	Lower
Overall survival	Comparable	Comparable
Disease-free survival	Comparable	Comparable

## New tools and methods

With advances in engineering and technology, surgical robots, such as the da Vinci robotic system, are continually improved. Together, new ideas and innovative methods, such as the suprapubic surgical approach, single-port robotic colectomy, intracorporeal anastomosis and the use of tracers, are applied in the clinic. These new changes have demonstrated promising potential in robotic colonic resection.

### Outcomes using the da Vinci Xi system

The da Vinci Xi (dVXi) surgical system is the fourth generation of surgical robot from Intuitive Surgical, Inc. (Sunnyvale, CA, USA), which attracted the attention of surgeons as soon as it was introduced to the market. The versatility of this new system, including integrated table motion, more sophisticated arms, and complex imaging units, enables it to perform a wide range of colonic procedures, from complex multiquadrant colectomies to intracorporeal anastomosis in a narrow space. The dVXi is also flexible in the complete mobilization of the colonic splenic flexure. Research investigating surgical outcomes of the dVXi has been reported continuously in recent years (Table 5). Use of the dVXi may result in shorter operative time, less estimated blood loss, and faster postoperative recovery than its predecessor, the da Vinci Si (dVSi), shorter length of hospital stay, and shorter duration of postoperative ileus than the laparoscopic approach. Long-term outcomes using this system, however, are currently lacking.

**Table 5:** Studies of surgical outcomes of da Vinci Xi

Authors	Publication year	Study type	Period	The term	Sample size		Surgery	Clinical outcomes*
Hill et al. [29]	2020	Retrospective cohort	2018.1–2019.3	Short-term	dVXi 41	dVSi 52	Sigmoidectomy or low anterior resection	Shorter operative time
Fleming et al. [30]	2020	Prospective study	2016.6–2019.7	Short-term	dVXi 100	–	Colectomy	Safe and feasible
Huang et al. [31]	2019	Retrospective cohort	2011.12–2017.10	Short-term	dVXi 60	dVSi 120	Colectomy	Lower rate of diverting ileostomy, shorter operative time, less estimated blood loss, and faster postoperative recovery
Beltzer et al. [32]	2019	Retrospective cohort	2013–2018	Short-term	dVXi 60	Laparoscopy 46	Sigmoidectomy of diverticulitis	Shorter duration of postoperative ileus
Liang et al. [33]	2019	Retrospective description	2015.1–2017.12	Short-term	dVXi 104	–	Left colectomy	Dexterous dissection during the mobilization of splenic colonic flexure
Protyniak et al. [34]	2018	Retrospective cohort	2014–2016	Short-term	dVXi 26	dVSi 44	Sigmoidectomy or low anterior resection	Comparable operative time, bleeding, and postoperative complications
Jimenez-Rodriguez et al. [35]	2018	Retrospective cohort	2015–2017	Short-term	dVXi 15	Laparoscopy 8	Total abdominal colectomy	Shorter length of hospital stay and similar operative time
Ngu et al. [36]	2017	Retrospective description	2015.3–2016.4	Short-term	dVXi 54	–	Single-docking left colectomy	Feasible

dVXi: da Vinci Xi. dVSi: da Vinci Si

^*^Da Vinci Xi compared with da Vinci Si or laparoscopy

### Optimization of the suprapubic surgical approach

**Fig. 2:**
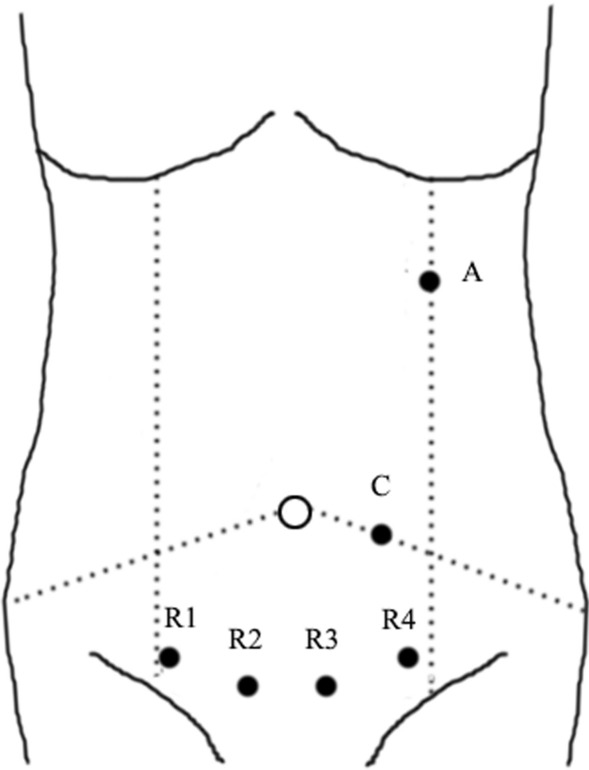
Port position of da Vinci Xi with suprapubic surgical approach in the right hemicolectomy. C: camera; R1-4: robotic instrument; A: assistant

Exploration of new surgical approaches, such as the optimization of the suprapubic method, is of clinical significance. A convenient surgical approach can increase the fluency of the operation and reduce collision of the robotic arms. The suprapubic surgical approach means colonic resection performed with horizontal linear placement of ports in the suprapubic area, especially applied in the robotic right hemicolectomy (Fig. 2). Hamilton et al. [37] reviewed technical and perioperative outcomes using the dVXi and dVSi systems with suprapubic port placement (SPPP) or traditional port placement (TPP) in 138 patients who underwent totally robotic right hemicolectomy (RRHC). The authors reported that SPPP had more advantages than TPP, with less console time and shorter hospital stay. Yeo et al. [38] developed a potentially universal SPPP strategy for robotic colectomy with complete mesocolic excision (CME) and central vascular ligation using the dVXi robotic system from cadaveric models. Lee et al. [39], from Korea, and Schulte Am Esch et al. [40], from Germany, separately described robotic right hemicolectomy using the suprapubic access strategy, and the perioperative effects were relatively satisfactory. The above studies reported optimistic short-term effects, and optimization of the suprapubic surgical approach may be widely used in the future when application conditions and long-term efficacy are further clarified.

### Application of single-port robotic surgery

**Fig. 3:**
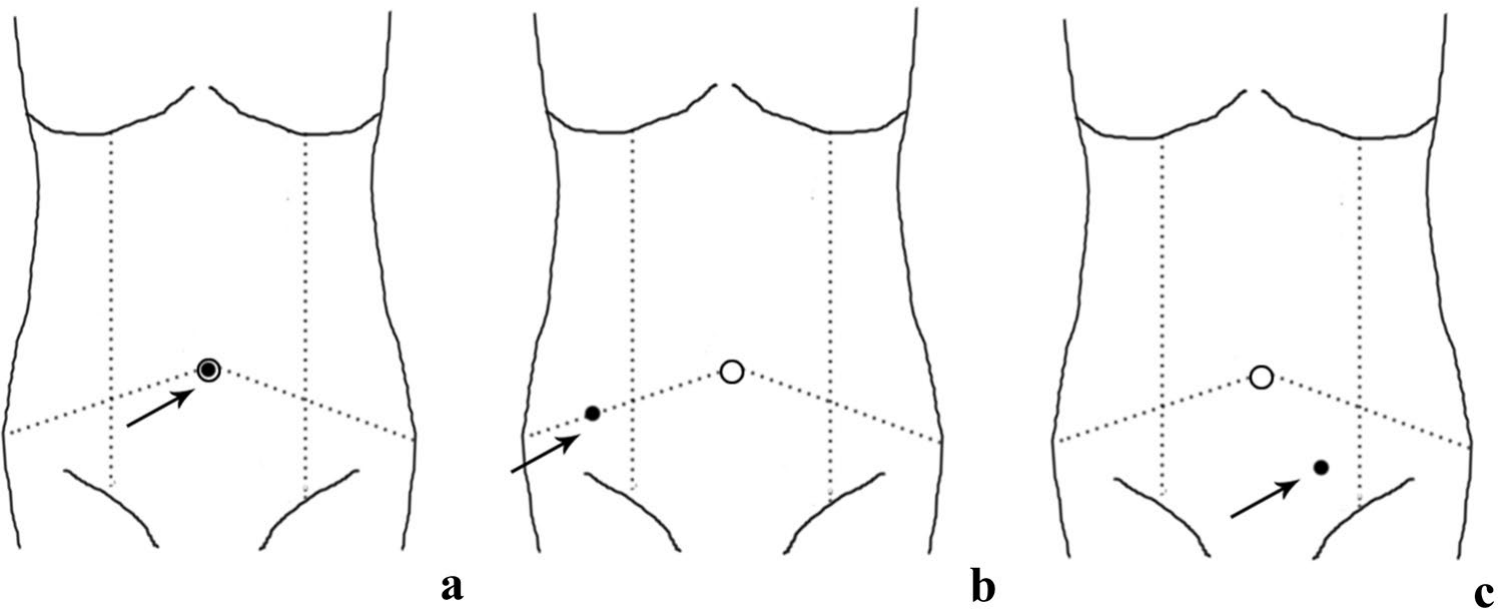
Port position of single-port robotic colectomy. **a** port position in single-port robotic total colectomy; **b** port position in single-port robotic left colectomy; **c** port position in single-port robotic right colectomy

Surgeons are attempting to reduce the number of robotic ports for better looking and faster recovery in robotic colonic resection. Single-port robotic (SPR) surgery has begun to be applied in the clinic, which just as its name implies, performs colonic resection through a single-incision with the assistance of robot (Fig. 3). From the United States, Juo et al. [41] completed 1 case of SPR total colectomy and reported that it was a feasible procedure associated with a small increase in operative time. In another study from the United States, Marks et al. [42] reported 2 cases, and Bae et al. [43] from Korea reported 23 cases of SPR left colectomy, indicating that this method was feasible and safe. Spinoglio et al. [44] successfully performed 3 cases of SPR right colectomies and completed intracorporeal anastomosis using the da Vinci single-site platform through a suprapubic incision. A systemic review [45] of current studies revealed that SPR surgery for colonic diseases was feasible and safe with acceptable perioperative outcomes (early postoperative complications 0–36.4% and hospital stay 2–9 days, comparable with those of multi-port robotic surgery). Presently, research investigating SPR colectomy is limited by small sample sizes. The results of larger-sample and longer-term studies, therefore, are eagerly anticipated.

### Use of intracorporeal anastomosis

Intracorporeal (IC) anastomosis of the intestinal canal during colectomy is a relatively new surgical method that changes the way in which the colon is pulled from the body through a surgical incision before anastomosis. This anastomosis method reduces the pulling of the intestinal tract to a certain extent, which may reduce postoperative complications. A systematic review and meta-analysis [46] from Italy found a higher rate of IC anastomosis in robotic right colectomy than in the laparoscopic group. Ngu et al. [47] from Singapore reported that robotic group recorded statistically shorter time for IC anastomosis, greater lymph node harvest and similar postoperative recovery and complication rate compared with laparoscopic group in right hemicolectomy with IC anastomosis. Some studies have verified the safety and feasibility of robotic IC anastomosis, and some reported shorter extraction site incisions, earlier bowel recovery, fewer complications, and lower rate of conversion, anastomotic leakage, surgical site infection, and incisional hernia, but longer operative time in IC than extracorporeal (EC) anastomosis, which indicated that IC had broad prospects for application in colonic surgery (Table 6). There remains a lack of long-term results of IC versus EC in robotic colonic resection.

**Table 6:** Studies of clinical outcomes between intracorporeal and extracorporeal anastomosis

Authors	Publication year	Study type	Period	The term	Sample size		Surgery	Clinical outcomes*
					Intracorporeal	Extracorporeal		
WIDMAR et al. [48]	2020	Retrospective cohort	2013–2017	Short-term	67	97	Right colectomy	Shorter length of hospital stay and more incisional surgical site infections
Emile et al. [49]	2019	Meta-analysis	2010–2018	Short-term	2123	2327	Right colectomy	Decreased extraction site incisions, earlier bowel recovery, and fewer complications and rate of conversion
Al Natour et al. [50]	2019	Retrospective cohort	2012.2–2017.11	Short-term	57	57	Sigmoidectomy	Fewer conversion, extraction site hernias, and longer operative time
Kelley et al. [51]	2018	Retrospective description	2016.8–2017.3	Short-term	21	–	Right colectomy	Feasible, safe, efficacious, and oncologically acceptable
Jung et al. [52]	2015	Retrospective description	2007.5–2011.2	Long-term	162	–	Transverse colectomy	Safe and feasible
Lujan et al. [53]	2015	Retrospective study of prospective data	2009.6–2012.9	Short-term	52	–	Right colectomy	Safe and feasible

^*^Intracorporeal anastomosis compared with extracorporeal anastomosis

### Development of totally robotic surgery

The proportion of total robotic colectomies without laparoscopic assistance has recently increased. Studies investigating totally robotic colonic resection have reported safety and feasibility. A retrospective study from China by Liu et al. [54] included 64 cases of totally robotic right hemicolectomy (TRRH) and 128 cases of RARH and compared short- and long-term outcomes. The authors reported longer mean operative time, incision length, lower postoperative pain score and shorter time to pass flatus in the TRRH compared with RARH group. The 3-year OS and DFS rate were comparable between the two groups. Ozben et al. [55] completed a retrospective review of 37 patients undergoing totally robotic right-sided CME with a mean operative time of 289.8 ± 85.3 min, estimated blood loss of 77.4 ± 70.5 mL, 41.8 ± 11.9 harvested lymph nodes, and mean length of hospital stay of 6.6 ± 3.7 days. Scotton et al. [56] reported a longer operative time and faster bowel function recovery and tolerance to solid diet in CME with totally robotic right colectomy versus hybrid robot-assisted right colectomy. However, the difference was not statistically significant.

The superiority of this method, however, needs further confirmation. Meanwhile, research investigating long-term efficacy is urgently needed. Totally robotic surgery with intracorporeal anastomosis may replace the existing hybrid robot-assisted extracorporeal anastomosis as the mainstream surgical approach in the future.

### Use of tracers

In recent years, the use of tracers in surgery has changed the face of colectomy, and indocyanine green (ICG) is the most commonly used tracer in colonic resection. ICG, a fluorescent tricarbocyanine compound, which rapidly binds to plasma proteins when injected intravenously, can produce fluorescence under infrared excitation. Currently, research investigating ICG assessing blood supply of anastomotic stoma to prevent anastomotic leakage is increasing, especially in laparoscopic colorectal surgery. In a systematic review and meta-analysis of intraoperative anastomotic testing in colorectal surgery from Treviglio Hospital (Lombardy, Italy), Rausa et al. [57] suggested that using ICG for blood supply assessment may reduce the anastomotic leakage rate. Yang et al. [58] reported that the use of ICG facilitated the delineation of the vascular anatomy, and Munechika et al. [59] demonstrated that high ligation of the inferior mesenteric artery for descending colon cancer under ICG fluorescence imaging was safe and effective in a pilot study. Van den Bos et al. [60] reported that both subjective and measured fluorescence intensity of ICG appeared to be related to anastomotic leakage in a clinical pilot study including 30 patients undergoing either laparoscopic or robotic anastomotic colorectal surgery.

Except for the assessment of vascularization of the colic stump, ICG can also be used as a tracer for lymph nodes in colectomy, which helps complete lymph node dissection more efficiently. Park et al. [61] performed a retrospective study and found that real-time ICG fluorescence imaging of lymph nodes may improve the performance of radical D3 lymph node dissection during laparoscopic hemicolectomy for advanced right-sided colon cancer.

Regrettably, this type of research investigating ICG in robotic colectomy is relatively rare. “Firefly” technology, integrated on the dVXi, enables tracers, such as ICG, to complete the assessment of colon perfusion and identification of lymph nodes more efficiently. Therefore, it is foreseeable that tracers will play an increasingly important role in colonic resection, and we believe that many reports describing the value of ICG in robotic colectomy are forthcoming.

## Learning curve of robotic surgery

The learning curve represents the number of cases for surgeons to achieve plateau performance through a new procedure. Pernar et al. [62] found that 19–128 cases of robotic colorectal operation were needed for surgeons. Shaw et al. [63] made a conclusion after retrospectively reviewed 62 patients that overall complications were reduced after first 15 cases. Symer et al. [64] reported that iatrogenic complications were reduced after surgeons completed 27 cases of robotic colorectal resection in a study including 2763 procedures. Gerbaud et al. [65] further reported that a surgeon, who was experienced in laparoscopic surgery, may not cause any increase on the morbidity rate of complications when started to perform robotic right colectomy with intracorporeal anastomosis. It can be seen from the above studies that, for surgeons, the learning curve of robotic surgery was relatively short, which was convenient for rapid clinical application.

## Surgical robots from multiple manufacturers

Since the turn of the century, robot applied in colonic surgery has been synonymous with the da Vinci robotic surgical system. The introduction of new robotic platforms will grow considerably in the near future as several manufacturers are active in the developing stages of robotic systems.

"MicroHand S", a surgical robot from China, has entered clinical trials. Some studies have also reported good performance and application prospects. Yi et al. [66] reported 10 surgical procedures with the assistance of MicroHand S without intraoperative complications or technical problems. Luo et al. [67] retrospectively analyzed 45 patients with sigmoid colon cancer who underwent MicroHand S or da Vinci robotic-assisted surgery. In patients with sigmoid colon cancer, the Da Vinci surgical system did not demonstrate obvious clinical advantages compared with the MicroHand S surgical system. However, the MicroHand S surgical platform demonstrated advantages in terms of the hospitalization cost and length of postoperative bedtime. The outcome of this study indicated that the MicroHand S surgical system may have good prospects for application in surgical fields in China.

The novel Senhance robotic system (TransEnterix Surgical Inc., Morrisville, NC, USA) has been used in a variety of applications in Europe and approved for limited clinical use in the United States. In a study from Germany, Darwich et al. [68] collected 12 patients who underwent sigmoid resection using the Senhance surgical robotic system and confirmed that this robotic system can be used safely in sigmoid resection for diverticular disease after adequate training. Samalavicius et al. [69] performed a prospective analysis of the first 100 robotic surgeries using the Senhance robotic system in Lithuania, and reported that this robotic system was feasible and safe in general surgery.

In addition to the MicroHand S and Senhance systems, “Hugo RAS” from Medtronic Inc. (Dublin, Ireland) and “Versius” from CMR Surgical Ltd. (Cambridge, United Kingdom) have demonstrated promising potential in clinical applications. The emergence of surgical robots from multiple manufacturers is bound to change da Vinci’s market share and the cost of surgery is expected to decrease, which ultimately benefits patients.

## Dissenting opinions regarding robotics

Although most studies have reported that robotic-assisted colectomy yielded equal or better surgical outcomes than the laparoscopic approach, a study from Johns Hopkins University School of Medicine (Baltimore, MD, USA) by Lo et al. [70] reached a different conclusion. The authors conducted a retrospective study of prospectively collected data to examine the impact of minimally invasive surgical approaches in frailty (defined as a loss of physiological reserve and association with adverse health outcomes, including disability, hospitalization, and mortality) with colon cancer. A total of 37,977 colectomies, performed between 2012 and 2016, were included and the primary outcome measure was 30-day postoperative complications. The study concluded that frailer patients experienced increased complication rate and were more likely to develop major complications. Results of this study serve as a reminder to be cautious when performing robotic colon surgery on certain subsets of the population, such as frail individuals.

## Summary

Advances in the development of minimally invasive surgery and the application of surgical robots with ergonomic advantages in colonic resection have afforded several benefits to patients. Comparative studies investigating the efficacy of robots and laparoscopies remain the focus of research in colon surgery. Simultaneously, the application of the optimized suprapubic surgical approach, single-port robotic surgery, robotic intracorporeal anastomosis, totally robotic surgery, and tracers for vessels and lymph nodes are gradually popularized in colonic resection. The introduction of new surgical robots from multiple manufacturers will reduce the burden of healthcare cost and bring a new look to the field of colonic surgery. With innovative approaches and emerging robotic technologies, we believe that robotic colonic resection will have good application prospects.
